# Undiagnosed myotonic dystrophy: A case report and literature review

**DOI:** 10.3892/mi.2023.106

**Published:** 2023-08-29

**Authors:** Tomonori Yamada, Natsumi Fukano, Kentaro Kai, Yoshihide Kuribayashi, Mika Jikumaru, Satoshi Eto, Yasushi Kawano

**Affiliations:** 1 Department of Obstetrics and Gynecology, Faculty of Medicine, Oita University, Yufu, Oita 879-5593, Japan; 2 Department of Anesthesiology and Intensive Care Medicine, Faculty of Medicine, Oita University, Yufu, Oita 879-5593, Japan; 3 Department of Neurology, Faculty of Medicine, Oita University, Yufu, Oita 879-5593, Japan

**Keywords:** adnexal torsion, genetic testing, myotonic dystrophy, undiagnosed disease, weaning failure

## Abstract

Myotonic dystrophy (MD) is an autosomal dominant disorder primarily characterized by myotonia. The present study describes the case of a 42-year-old woman who was transferred to the authors' department with acute abdomen and restrictive respiratory failure. Computed tomography revealed a 15-cm right ovarian tumor and atelectasis. An abdominal right salpingo-oophorectomy was performed under general anesthesia. She was then extubated after surgery; however, shortly thereafter she was re-incubated due to poor oxygenation and was then moved to the intensive care unit (ICU) for a further analysis of weaning failure. During her stay in the ICU, weaning was attempted twice, but failed both times. The patient underwent a tracheotomy 7 days after surgery. Consultation with a neurologist suggested possible MD. Following genetic testing, type I MD with ~700-1,100 cytosine-thymine-guanine repeats in the dystrophia myotonia protein kinase gene was confirmed. The patient was then transferred to a specialty hospital at 2 months after surgery. On the whole, the case described herein suggests that clinicians need to become familiar with this disease as a differential diagnosis for post-operative weaning failure.

## Introduction

Myotonic dystrophy (MD) type 1 (MD1) is an autosomal dominant multisystem disorder clinically characterized by skeletal muscle weakness, myotonia, cardiac conduction abnormalities, cataracts and other abnormalities (1). MD1 results from an expansion of a cytosine-thymine-guanine (CTG) trinucleotide repeat in the 3'-untranslated region of the dystrophia myotonia protein kinase (*DMPK*) gene (2). A study in 2021 based on the newborn screening program in New York State reported DM1 prevalence of 4.76 per 10,000 births or 1 in 2100 births (3). The length of the CTG repeat expansion is moderately associated with disease severity (4). In the case that the number of repeats is high, MD1 can be diagnosed based on characteristic symptoms and a positive family history. The anesthetic management of patients with MD1 warrants careful attention due to individual differences in reactions to certain anesthetics and the multisystem effects of the disease (5). However, in the case that the number of repeats is low, MD1 may progress unnoticed by patients and their families. General anesthesia in patients with undiagnosed MD1 can cause post-anesthesia complications.

Difficult-to-wean patients are those who fail initial weaning and require up to three spontaneous breathing trials (SBTs) or as long as 7 days from the first SBT to achieve successful weaning (6). In the surgery room, airway obstruction, inadequate ventilation and oxygenation, and the clearance of secretions can present difficulties in extubating patients. In a previous multicenter, prospective study of patients in the intensive care unit (ICU), the incidence rate of difficult-to-wean cases was found to be 39% of the patients who received mechanical ventilation for at least 12 h (7). Patients in this category should be identified for etiologies, such as respiratory, cardiac, psychological, ventilator circuit and nutritional issues, as well as ICU-acquired weakness (8). This workup process can sometimes be difficult if the disease etiology is unknown.

The present study describes the case of a woman who was diagnosed with MD1 for the first time following surgery that was triggered by post-operative weaning failure from mechanical ventilation.

## Case report

**Figure 1:**
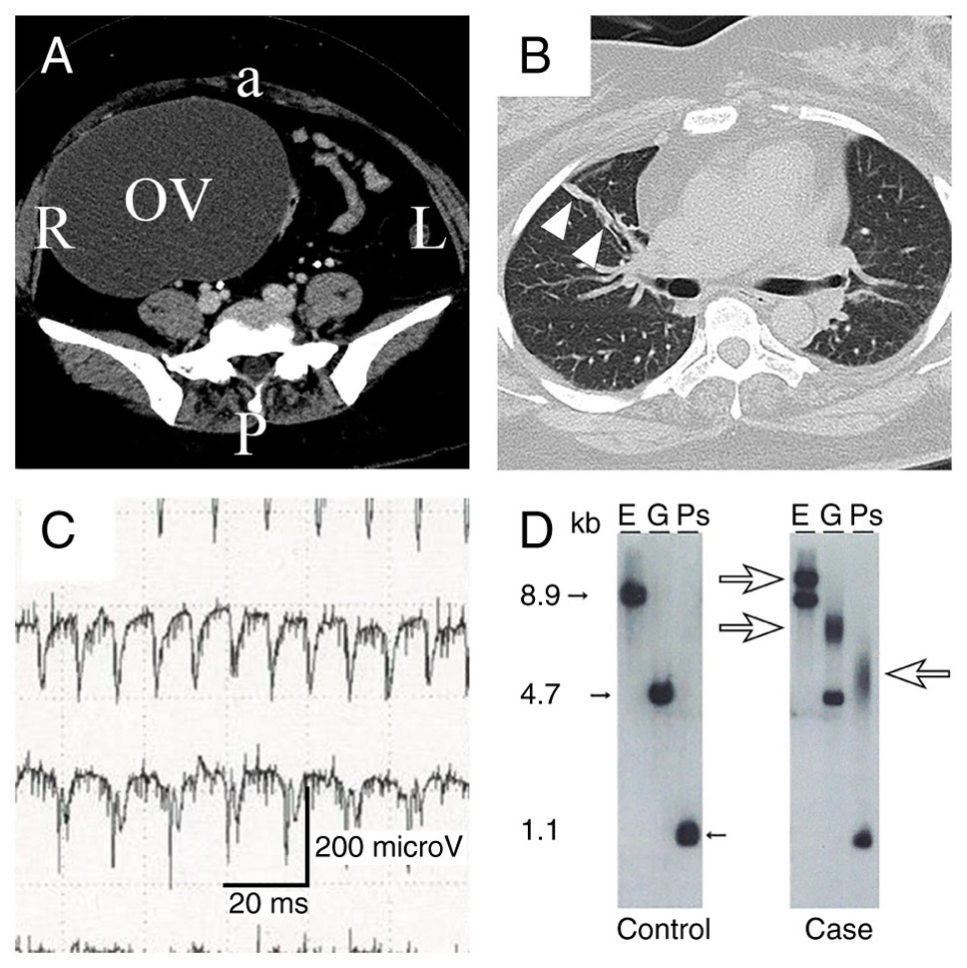
Results of pre-operative imaging and tests for definitive diagnosis. (A) Pelvic computed tomography scan illustrating a cystic right ovarian tumor (OV). a, anterior; P, posterior; L, left; R, right. (B) Chest computed tomography scan illustrating right pulmonary discoid atelectasis (arrowheads). (C) Needle electromyography of the biceps indicating myotonic discharge. (D) Southern blotting. The left panel demonstrates the *DMPK* gene from a healthy control. The right panel demonstrates the *DMPK* gene from the patient described herein. Small arrows (closed) in lanes E, G and Ps indicate normal repeat bands ranging from 5 to 34. Large arrows (open) in lanes E, G and Ps indicate expansion repeat bands ranging from 700 to 1,100 kb. kb, kilobyte; E, *EcoR*I; G, *Bgl*I; Ps, *Pst*I.

A 42-year-old non-gravid Japanese woman visited Nakatsu Gastrointestinal Hospital (Nakatsu, Japan) complaining of sudden-onset lower abdominal pain. Contrast-enhanced computed tomography revealed the torsion of the right ovarian tumor and pulmonary discoid atelectasis (Fig. 1A and B). She was referred to the gynecological department of Nakatsu Municipal Hospital and scheduled for surgery. However, a pre-operative pulmonary function test revealed severe restrictive respiratory failure (the vital capacity was 30% and forced expiratory volume in the first second was 103%). Arterial blood gas analysis revealed respiratory acidosis (pH 7.25; partial pressure of carbon dioxide, 68.2 mmHg; and bicarbonate, 28.9 mmol/l). Her chest radiograph, electrocardiogram and laboratory test results did not reveal any abnormalities. The patient was then transferred to Oita University Hospital (Oita, Japan) for high-risk emergency surgery. Of note, 3 years prior, she had been diagnosed with obesity-related restrictive lung disease by a primary care doctor and was advised to lose weight (her body mass index was 33.6 kg/m^2^ at the time of the present analysis). She had undergone a laparoscopic myomectomy at 36 years of age and seven consecutive failures of artificial inseminations. She underwent an abdominal right salpingo-oophorectomy, which was converted from laparoscopy owing to severe pelvic adhesions.

Anesthesia was induced with propofol (1.7 mg/kg) and remifentanil [0.2 µg/kg/min, intravenously (i.v.)]. Tracheal intubation was facilitated with rocuronium bromide (0.7 mg/kg, i.v.), and anesthesia was maintained with 5% desflurane in 70:30 air: O_2_ and remifentanil infusion (0.25 µg/kg/min). Ephedrine (4 mg/dose, i.v.) and phenylephrine (0.1 mg/dose, i.v.) were used to reduce any hypotensive response during surgery. The patient was mechanically ventilated to maintain the lower limit of normal end-expiratory CO_2_ (36-37 mmHg). She was extubated following surgery, but was promptly reintubated owing to respiratory failure that was unresponsive to manual ventilation. She was then transferred from the operating theater to the ICU for post-operative respiratory management. On post-operative day (POD)1, she was extubated again and supported by non-invasive positive pressure ventilation. On POD2, her respiratory support was gradually decreased from high nasal flow through a face mask to a nasal cannula. On POD3, she was moved to the general ward. However, owing to respiratory failure at 9 h after transfer, the patient was reintubated for the second time and returned to the ICU. On POD5, she was extubated again, and bi-level positive airway pressure support was initiated following consultation with a pulmonologist with the assessment of obesity-related respiratory failure. On POD6, regardless of the cricothyroid membrane puncture-guided tracheotomy, the sputum retention of the patient remained uncontrolled, and she was intubated for the third time. She was thus diagnosed as a difficult-to-wean patient and a tracheostomy was performed on POD7, followed by further workup.

The consulting neurologist suspected MD based on the patient's characteristic symptoms. Her facial and limb muscles were weak (manual muscle test grade was 4/5) (9), her tongue was atrophied, and she was obese. Percussion myotonia was observed. Her medical history obtained through her written messages and from her family suggested that her skeletal muscle weakness had gradually progressed from adolescence onwards. At the age of 20, the patient could not inflate a balloon, and at the age of 35, she could not open the lid of a plastic bottle. At the most recent follow-up, she could not walk for 100 m without resting. Her family history was not definite; however, her mother had a similar facial appearance and cognitive dysfunction. The patient's mother was a potential carrier of an abnormal *DMPK* gene.

A needle electromyography revealed myotonic discharge in the biceps (Fig. 1C). Following genetic counseling, molecular genetic testing for the *DMPK* gene was performed, which revealed a CTG repeat length of 700-1,100 bp (Fig. 1D). The genetic testing was performed by LSI Medicine Corporation (Inspection code 02820, Tokyo, Japan). Finally, the patient was diagnosed with MD1. She continued dysphagia rehabilitation for weaning from the ventilator in hospital. Continuous dysphagia rehabilitation resulted in recovery to ventilation with minimum support [pressure support (PS)/continuous positive airway pressure; fraction of inspired oxygen, 0.3; positive end-expiratory pressure, 6 mmHg; PS, 8 mmHg] on POD39. The patient was then transferred to Nishibeppu Hospital (Beppu, Japan) on POD48.

## Discussion

The present study describes a case of MD, with two key clinical issues. First, MD1 may become clinically evident following post-operative weaning difficulty in patients with a history of uneventful general anesthesia. Second, MD1 may be predicted much at an earlier stage based on a combination of pre-operative respiratory failure and a gynecological history.

Several factors can cause an imbalance between respiratory muscle strength and the workload of breathing, resulting in weaning difficulties in the ICU. These factors comprise respiratory or ventilatory, cardiac, psychological, ventilator circuit-related and nutritional issues (8,10). A summary of the reported cases of MD1 diagnosed following post-operative weaning failure is presented in Table I (11-14). The present study also conducted a literature review regarding undiagnosed MD by searching PubMed/MEDLINE and Google Scholar. The terms ‘respiratory failure’, ‘myotonic dystrophy’ and ‘undiagnosed myotonic dystrophy’ were used to search the literature in English without publication date filters. Patients from all four studies required long-term post-operative ventilator management and tracheostomy, resulting in a poor prognosis in some cases (11-14). None of the patients were identified to have a family history of MD1, although similar findings were later demonstrated in the patients' families by consultant neurologists (11,12). It should be noted that only the case described herein had a history of uneventful general anesthesia. This is one of the reasons that a certain amount of time was needed to reach a definite diagnosis of MD1.

Previous studies have demonstrated that tumorigenesis, including gynecological benign and malignant tumors, is a key clinical manifestation of MD1. A previous meta-analysis included five studies comprising 2,779 patients and revealed that the standardized incidence ratio for endometrial cancer was 7.48 [95% confidence interval (CI), 4.72-11.8] and 5.56 (95% CI, 2.99-10.3) for ovarian cancer (15). The UK Myotonic Dystrophy Patient Registry reported that the incidence of benign gynecological tumors ranged from 6.1 to 37.6% for uterine myoma and 3.5% for ovarian cysts (16). Furthermore, the female sex was significantly associated with benign tumors in multiple organs (16). Thus, patients with MD, particularly females, have a higher chance of undergoing general anesthesia surgery than the general population. Therefore, clinicians should pay attention not only to common clinical manifestations, such as musculoskeletal weakness, cardiac defects and early cataracts, but to the tumorigenesis of MD1 as well.

In conclusion, the present study describes the case of a woman with post-operative weaning difficulty with unrecognized MD1. The risk of general anesthesia for patients with MD1 is widely known. Therefore, many individuals with known MD1 carry a card to alert authorities of their risk regarding anesthesia. Conversely, a history of uneventful general anesthesia may be a pitfall in the differential diagnosis of patients with weaning difficulties. Furthermore, clinicians need to be more familiar with the variable manifestations of MD1 in multiple organs other than neuromuscular symptoms. In patients with certain risk factors (such as a history of gynecological tumors or respiratory failure), timely identification and evaluation of these risk factors would help to ensure safer and more effective treatment outcomes. Furthermore, based on the findings of the present case report, further prospective studies are warranted to systematically evaluate the risks and influencing factors that the majority of patients with MD1 face regarding post-extubation difficulty, providing more substantial evidence for clinical practice.

## Figures and Tables

**Table I:** Reported cases of myotonic dystrophy 1 diagnosed after surgery triggered by post-operative weaning failure.

Study no.	Authors	Year of publication	Age, years	Diagnosis	Type of surgery	Previous history of surgery	*DMPK* status	Symptoms and physical signs	Outcome	(Refs.)
1	Fossen and Gjerstad	1986	34	Atonic bleeding	Abdominal hysterectomy	C-section (local anesthesia)	N/A	Bilateral ptosis, facial and distal limb muscle weakness, myotonic grip, percussion myotonia	Recovery in the 6th week	(11)
2	Gupta *et al*	2009	32	Adnexal mass	Exploratory laparotomy to rule out cancer	None	Expansion (160,900)	Temporal flattening and frontal balding, scoliotic deformity of dorsal spine, muscle spasm	Dead of cardio-pulmonary arrest in ICU on the 391st day	(12)
3	Gómez Hernández *et al*	2016	73	Thymoma and thyroid cancer	Cervicotomy and partial sternotomy	Cataract surgery and basal cell carcinoma surgery (local anesthesia)	Expansion	Baldness, dropping eyelids, early cataracts, muscle weakness, swallowing difficulties	Succumbed due to asphyxia in the hospital in the 1st month	(13)
4	Ota *et al*	2019	20	Appendiceal endometriosis	Laparoscopic biopsy and appendectomy	N/A	Expansion (400 to 450)	Long and narrow facies with atrophic temporalis	Alive and discharged on the 69th day	(14)
5	Present study	2023	42	Adnexal torsion	Abdominal salpingo-oophorectomy	Laparoscopic myomectomy (general anesthesia)	Expansion (700 to 1,100)	Facial and limb muscles weakness, tongue atrophy, percussion myotonia, obesity	Alive and transferred on the 48th day	

N/A, not available.

## Data Availability

The datasets used and/or analyzed during the current study are available from the corresponding author on reasonable request.
